# Aseptic Surgery

**Published:** 1896-12

**Authors:** 


**Aseptic Surgery.** By CHARLES BARRETT LOCKWOOD. Pp. xiv., 233. Edinburgh: YOUNG J. PENTLAND. 1896.

This work is a compilation of "notes" which were published in the *St. Bartholomew's Hospital Journal*, and although, as the author truly and modestly says in the introduction, they have "no pretensions to completeness," they may be read with profit by students and medical men as an introduction to the science of which they treat. The work is divided into four parts: (1) The principles upon which aseptic surgery is founded; (2) the distribution of bacteria; (3) disinfectants and antiseptics; and (4) their method of application to surgical practice. The author states clearly and forcibly those views in which he believes, and by the application of which he has worked so well. There is, however, a somewhat unnecessary repetition through the book—*e.g.* the footnote on p. 167 is incorporated in the text on p. 164. The printing is clear and apparently free from errors, the binding is neat, and there is a most complete index. We can recommend the book as a sound and practical elementary treatise.

